# Oral Health, Temporomandibular Disorder, and Masticatory Performance in Patients with Charcot-Marie-Tooth Type 2

**DOI:** 10.1155/2013/425651

**Published:** 2013-12-10

**Authors:** Rejane L. S. Rezende, Leonardo R. Bonjardim, Eduardo L. A. Neves, Lidiane C. L. Santos, Paula S. Nunes, Catarina A. Garcez, Cynthia C. Souza, Adriano A. S. Araújo

**Affiliations:** ^1^Programa de Pós-Graduação em Ciências da Universidade Federal de Sergipe-UFS, Rua Cláudio Batista S/N Bairro Sanatório, 49.060-100 Aracaju, SE, Brazil; ^2^Departamento de Ciências Biológicas da Faculdade de Odontologia de Bauru-FOB/USP, Avenida Otavio Pinheiro Brizola, 9-75, 17012-901 Bauru, SP, Brazil; ^3^Departamento de Fisiologia da Universidade Federal de Sergipe-UFS, Avenida Marechal Rondon, S/N Jardim Rosa Elza, 49.100-000 São Cristovão, SE, Brazil

## Abstract

*Background*. The aim of this study was to evaluate the oral health status of temporomandibular disorders (TMD) and bruxism, as well as to measure masticatory performance of subjects with Charcot-Marie-Tooth type 2 (CMT2). *Methods and Results*. The average number of decayed, missing, and filled teeth (DMFT) for both groups, control (CG) and CMT2, was considered low (CG = 2.46; CMT2 = 1.85, *P* = 0.227). The OHIP-14 score was considered low (CG = 2.86, CMT2 = 5.83, *P* = 0.899). The prevalence of self-reported TMD was 33.3% and 38.9% (*P* = 0.718) in CG and CMT2 respectively and for self-reported bruxism was 4.8% (CG) and 22.2% (CMT2), without significant difference between groups (*P* = 0.162). The most common clinical sign of TMD was masseter (CG = 38.1%; CMT2 = 66.7%) and temporalis (CG = 19.0%; GCMT2 = 33.3%) muscle pain. The geometric mean diameter (GMD) was not significantly different between groups (CG = 4369; CMT2 = 4627, *P* = 0.157). *Conclusion*. We conclude that the CMT2 disease did not negatively have influence either on oral health status in the presence and severity of TMD and bruxism or on masticatory performance.

## 1. Introduction

The Charcot-Marie-Tooth (CMT) disease is a progressive neurological disorder that affects the peripheral nerves, causing weakness, muscle atrophy, and loss of sensitivity, especially in distal segments of the upper and lower limbs, with a highly variable clinical course [1–4]. It is part of the group of hereditary neuropathies and has an estimated prevalence of 37/100.000 individuals [5]. Trophic alterations like *pes cavus*, atrophy of the distal leg, and deformities of the toes are part of the clinical manifestations more frequently associated with CMT [2].

The CMT disease is classified according to the nerve structure which is primarily affected: myelin sheath or axon [2, 5–8]. The subclassing is based on the genes involved [6]. The most common form of CMT disease is type 1, also called demyelinating, where the conduction velocity is quite low. The CMT disease type 2 shows a prevalence of approximately 3 to 12/100.000 and is known as the axonal form of the disease with nerve conduction velocity either normal or slightly reduced [3, 4].

The first symptoms of CMT usually appear in childhood or adolescence, and the motor impairment is predominant, occurring in the lower extremities, slowly evolving towards distal-proximal, which can also affect the upper extremities [2, 9–11]. Clinical signs arise on the feet, on the legs, and later on the hands. As the disease progresses, the person may have other clinical signs, such as scoliosis, *pes cavus*, and tremor of the hands [12]. Reports of respiratory dysfunction such as diaphragmatic weakness have been described [13–16]. Sensory hearing loss can also occur [17, 18], paresis of the vocal cords that may be associated with a more severe variant of the disease [19]. Besides, it was found that this disease can also affect the quality of sleep [20].

However, to the best our knowledge, studies investigating the influence of this disease in the orofacial region are scarce, with the only case reports investigating the presence of trigeminal neuralgia [21] and self-reported mastication [22] in Charcot-Marie-Tooth disease. Although CMT disease is a distal neuropathy preferably, by having a progressive characteristic, the orofacial region may be affected, leading to an impairment of motor function which could compromise chewing. Besides, atrophy and weakness of the intrinsic muscles of the hand, it could also contribute to disability in the oral hygiene of these individuals.

So, this study aimed to characterize the condition of oral health and its impact on quality of life as well as to evaluate signs and symptoms of temporomandibular disorders, bruxism, and masticatory performance in individuals with CMT2 disease from a multigenerational family.

## 2. Methods

### 2.1. Sample and Ethical Consideration

The present study was conducted from August 2010 to February 2012 to evaluate a group of individuals presenting Charcot-Marie-Tooth type 2 disease according to oral health and its impact on quality of life. It also verified the signs and symptoms of TMD, bruxism self-reported, and masticatory performance. All these individuals were of the same family and presented the neuropathy transmitted with an autosomal dominant inheritance pattern. The members of this family live in the city of Tobias Barreto, that is, situated 180 km from Aracaju, Sergipe State, Brazil.

The diagnosis of CMT2 was done by a neurologist according to previously described by Neves and Kok [2].

Additionally, in the present study were included a control group (CG) which was constituted of individuals living in the same city but without CMT2 disease; this group was too evaluated according to according to oral health and its impact on quality, signs and symptoms of TMD, bruxism self-reported, and masticatory performance.

The present study was approved by the Research Ethics Committee of Federal University of Sergipe (CAAE: 62350000107-10). All participants of the study signed a written consent groups and after they were evaluated for the same examiner as explained below.

### 2.2. Study Design

#### 2.2.1. Oral Health

Determination of decayed, missing, and filled teeth (DMFT) for permanent teeth was done by visual examination according to the criteria set by the World Health Organization [23].

#### 2.2.2. Salivary Parameters

*Flow and pH Salivary*. The determination of salivary flow was made directly by reading the total volume of stimulated saliva of the subject obtained in five minutes. For this, the individual chewed continuously for 6 min a gum containing no sugar in its composition. The whole saliva produced during the first minutes of stimulation was swallowed or expelled. During the subsequent 5 minutes, the individual continued to chew the gum, which stimulated the salivary flow rate and was measured and calculated as mL/min. The basal physiological pH was measured with pH meter immediately after the stimulated saliva was collected to score acid, neutral, or basic [24].

*The Buffering Capacity of the Saliva (CTS) by Titration*. The determination of the CTS was performed by titration, measuring the volume of lactic acid 0.1 N required to reduce salivary pH 6.9 to 3.7 (turning point of the methyl orange). The indicator is yellow-orange in pH 6.9 and pH 7 in pink. 2 mL of stimulated saliva was placed in an Erlenmeyer flask, along with 1 drop of methyl orange, and poured in saliva dropwise, and lactic acid 0.1 N is placed in a burette until a pink color (turning of methyl orange). Close the burette and then read the volume of lactic acid 0.1 N spent. The value of the CTS is the result of the volume of lactic spent multiplied by 100. Individuals were classified according to the risk of caries according to the following groups: (1) patients moderately susceptible to dental caries (CTS = 40), (2) patients resistant to dental caries (CTS > 40), and (3) patients susceptible to dental caries (CTS < 40) [25].

#### 2.2.3. Evaluation of Profile Impact of Oral Health (OHIP-14)

The impact produced by oral health status on quality of life of individuals was assessed by the questionnaire profile impact of oral health (OHIP-14), whose Portuguese version was validated by de Oliveira and Nadanovsky [26].

This instrument consists of 14 questions divided into seven domains: functional limitation, physical pain, psychological discomfort, physical disability, psychological disability, social disability, and handicap. The questions were answered on a Likert scale with the following scores: never = 0, rarely = 1, sometimes = 2, often = 3, and always = 4, and the results were evaluated dichotomously. Then, the sum of the responses was performed to each question to obtain the total score OHIP-14 which can vary from 0 to 56 [27]. The higher the score, the greater the impact of oral conditions on quality of life.

#### 2.2.4. Evaluation of Signs and Symptoms of Temporomandibular Disorder (TMD)

A self-report questionnaire was used to assess subjective symptoms according to Conti [28]. This questionnaire consists of 10 questions (presence/absence of facial pain, bruxism, headache, difficulty in chewing, pain or limitation of mouth opening, and joint noise) that classify individuals for the presence and severity of temporomandibular disorders. Each question could be answered with a “yes,” “sometimes,” or a “no.” Each “yes” answered received a score of 2 (two), “sometimes” score 1 (one), and “no” score 0 (zero). Then, the sum of the scores was performed and the individuals were classified as TMD Free (scores 0–3), Mild TMD (scores 4–8), Moderate TMD (scores 9–14), or Severe TMD (scores 15–23).

Clinical signs of TMD were obtained through a clinical examination based on Brazilian version of the research diagnostic criteria for diagnosis of TMD (RDC/TMD) [29, 30].

The clinical signs evaluated were quality and range of mandibular motion, presence of TMJ sounds, presence of TMJ pain, and the masticatory muscles pain on palpation following the criteria proposed by Dworkin and Leresche [29].

#### 2.2.5. Evaluation of Sleep Bruxism-American Academy of Sleep Medicine (AASM, 2005)

The diagnosis of sleep bruxism was based on the clinical and anamnestic criteria of the American Academy of Sleep Medicine [31]. For diagnosis, the subjects reported or were aware of tooth-grinding sounds or tooth-clenching during sleep and one or more of the following should be present:

1. abnormal wear of the teeth,
2. jaw muscle discomfort, fatigue, or pain and jaw locking upon awakening,
3. masseter muscle hypertrophy during voluntary forceful clenching.

#### 2.2.6. Assessment of Masticatory Performance

The tests were performed with a food simulant, called Optocal, similar to that advocated by Slagter et al. [32]. Before conducting the test the subjects were trained on masticatory movement and the use of mouthwash, so that only chewing and no swallowing happened. During testing, the subjects chewed the Optical tablet with 20 movements, unilaterally or bilaterally, with the number controlled by the examiner. After each bite, all material was dispensed in a plastic container covered with a polyethylene filter strainer and the patient was asked to rinse the mouth twice. The rinse water was also collected with the chewed material, while ensuring the removal of any residue. Then, the collected material was filtered through a set of seven stacked sieves (Bertel Indústria Metalúrgica Ltda, SP, Brazil) with apertures of 5.6, 4.0, 2.8, 2.0, 1.4, 1.0, and 0.71 mm, coupled in descending order of aperture size, and placed on an agitator for 5 minutes. The particles retained on each sieve were weighed on an analytical balance.

Based on the weight of the Optical retained on each sieve, the geometric mean diameter (GMD) of the ground particles was calculated using the weighted geometric mean using Excel spreadsheets (Microsoft). The GMD represents the index of performance/chewing efficiency, with a lower value obtained from a smaller GMD, indicating better MP [33].

### 2.3. Statistical Analysis

Data are expressed as mean ± standard deviation, absolute and relative values. Comparisons between groups were performed using two-sided unpaired Student's *t*-test and chi-square or Fisher exact test, with significance level set at 5%.

## 3. Results

Table 1 shows the general characteristics of the sample related to gender, age, and marital status with no significant differences found. Self-reported gingival bleeding was high in both groups (CG = 47.6%; Charcot-Marie-Tooth type 2 group (GCMT2) = 50.0%) with no significant difference (*P* = 0.882).

The average of decayed, missing and filled teeth (DMFT) is presented in Table 2. The mean values of DMFT were considered low for both groups (CG = 2.46; GCMT2 = 1.85), with no significant difference (*P* = 0.2274). For the control group there was a higher rate of filled teeth (2.82) and for the GCMT2 of decayed teeth (2.22).

The mean values of total salivary volume, salivary flow, pH, and buffer capacity between the groups are shown in Table 3. No significant difference between groups was found.

**Figure 1:**
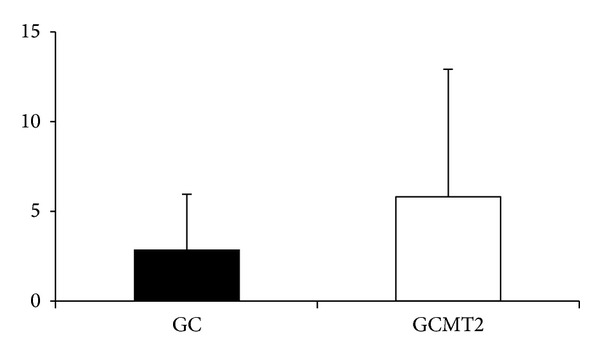
Mean values of OHIP-14 in control and CMT2 groups.

The mean scores OHIP-14 between groups are showed in Figure 1. The scores were low (GC = 2.86 ± 3.09, GCMT2 = 5.83 ± 7.10) and no significant difference between groups was found (*P* = 0.899).

Table 4 indicates the presence and severity of DTM according to the subjective symptoms reported by the subjects. In both groups, the most common finding was the absence of TMD (CG = 66.7%; GCMT2 = 61.1%) followed by mild TMD (CG = 28.6%; GCMT2 = 22.2%). No significant difference was found between groups (*P* = 0.718).

Table 5 shows clinical signs of TMD. The most common findings were pain in the masseter (CG = 38.1%; GCMT2 = 66.7%) and temporalis muscles (GC = 19.4%; GCMT2 = 33.3%). For all clinical signs evaluated, no difference was found between the groups, but a tendency of pain in masseter occurred to be significantly more common in GCMT2 (*P* = 0.075).

The frequency of sleep bruxism was low, being more common in CMT2 group (22.22%), however, without difference between groups (*P* = 0.162).

**Figure 2:**
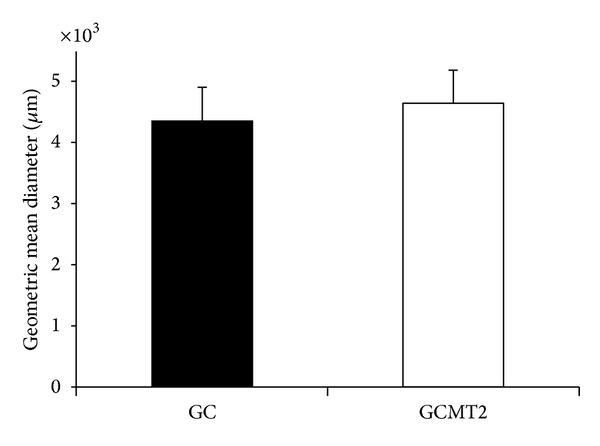
Mean values of geometric mean diameter (GMD) of the chewed particles between GC and GCMT2.

The geometric mean diameter (GMD) of the chewed particles is showed in Figure 2. The values of GMD were similar (GC = 4369 ± 521; CMT2 = 4627 ± 546.19), without difference between groups (*P* = 0.157).

## 4. Discussion

Oral health status was quantified using the DMFT score [23] as an effective tool used in epidemiological surveys to measure the oral health condition of a population. A low value of DMFT was observed, indicating a low frequency of decayed, missing, and filled teeth that did not differ between groups. This finding may influence directly and positively the quality of life (OHIP-14) of subjects with CMT2, which agrees with the study by Ide et al. [34], which found that those individuals who considered their oral health status as poor had a greater impact on quality of life assessed by OHIP. Furthermore, this same study showed a strong association between higher number of missing teeth and the highest score of the profile of the impact of oral health on quality of life [34].

The OHIP-14 used in this study was developed for older adults. However, this instrument can be used safely in adults and young people [35]. It was used because it is a method widely used in the literature review [36–39], which was validated for the Portuguese language [26].

The data presented here are pioneers in subjects with CMT2 which make difficult comparisons. However, it has been found that individuals with special needs tend to have poorer oral hygiene and periodontal problems and greater number of caries and missing teeth [40, 41]. Moreover, some studies in individuals with special needs, where manual dexterity is often compromised, similar to CMT2, show contradictory results.

A condition of poor oral health in individuals with Parkinson's disease (PD) related to general population has been verified [42, 43]. Hanaoka and Kashihara [44] found a greater number of lost teeth and caries and a high frequency of periodontal disease in patients with PD. However, some studies showed lower DMFT score compared to a group of individuals of the same age and without the disease [45, 46].

Although our study had shown a low prevalence of DMFT, the self-reported gingival bleeding was high in subjects with CMT2 which, however, did not differ from the control group. Perhaps the lack of manual dexterity to execute oral hygiene, was not the single factor that negatively impact on oral health of this population. So, this study investigated the salivary parameters.

Due to the importance of saliva in relation to caries prevention, the present study investigated some salivary parameters, such as the buffer capacity and salivary flow that were within the normal range for both groups, which may also have contributed to the low rate of DMFT. The salivary buffer capacity is an important factor in resistance to dental caries, and reduced salivary flow, which is usually associated with a low buffering capacity, can contribute to poor oral health [47].

In this study the mean pH of individuals with CMT2 was considered within the normal range. Previous studies have found a reduced salivary pH in subjects with dental caries [48–50]. Additionally, Farsi [51] found that the pH of saliva was the only parameter which was related to dental caries.

Furthermore, it should be noted that all individuals in the city where this study was conducted are supplied with fluoridated water which is also considered a preventive factor for dental caries [52].

Likewise, this study found that the frequency of signs and symptoms of TMD and the presence of sleep bruxism were not different between the control and CMT2 groups.

Related to self-reported symptoms of TMD, most individuals with CMT2 showed without TMD, followed by mild degree of TMD.

In a recent study in Brazil, through a validated telephone interview including 1230 people, it was found that at least one, two, and three or more self-reported symptoms occurred, respectively, in 39.2%, 17.6%, and 9.2% of the sample [53] which in part is similar to the findings of this study for individuals of CMT2.

The presence of clinical signs of TMD varied greatly in individuals with CMT2 (11.1–66.7%) and the presence of pain in the masseter and temporalis muscles was the most common findings; however, most of them were of mild degree similar to self-reported symptoms. Although, to our knowledge, no studies evaluated the presence of signs and symptoms of TMD in CMT, some case reports have shown the involvement of cranial nerves in CMT disease [54], especially the presence of trigeminal neuralgia [21, 22].

The pain has not been considered as a relevant symptom in individuals with CMT [55], although in this study pain in the masseter, of mild degree, was a common finding.

Because CMT is a neurological disease is more common the presence of neuropathic pain, such as trigeminal neuralgia, than musculoskeletal pain, such as the TMD which is corroborated by Gemignani et al. [56] who found that the nociceptive pain was more prevalent in CMT1A. The same authors, however, reported that it is expected that nociceptive pain such as musculoskeletal pain is less common than neuropathic pain in the CMT which is justified, in part, by the pathophysiology of the disease.

However, in a previous study using the same family evaluated in the present study, none of the individuals complained of neuropathic pain and, when present, sensory complaints were most often consistent with late onset nociceptive pain [2], corroborating our findings to pain in masseter muscle.

Masticatory performance measured by determining the geometric mean diameter (GMD) of the chewed particles did not differ between CG and GCMT2. These results corroborate a case study that found no impairment of chewing in a young woman with CMT [22].

One of the most common and reliable ways to evaluate the chewing is by analyzing the masticatory performance, which measures the distribution of chewed particles after a standard number of chewing cycles [57]. This tool was used in this study.

Some factors may reduce the masticatory performance as the decrease in salivary flow [58, 59], bite force [60, 61], the number of dental units [62], and the presence of TMD [63, 64].

In the present study, salivary flow was normal, the DMFT was considered low according to WHO, as well as the frequency of signs and symptoms of TMD. Moreover, atrophy and muscle weakness common in this disease affect more frequently the distal regions of the body. These findings may have contributed to a satisfactory masticatory performance in CMT2 subjects similar to that of a general population.

Thus, it can be suggested that the oral health condition, the signs and symptoms of TMD, and masticatory performance do not appear to be more impaired in individuals with CMT2 than the general population. However, longitudinal studies are necessary to follow individuals with CMT2 for a longer period of time to verify if the progress of this disease may, in the future, negatively impact oral health and masticatory function.

## Figures and Tables

**Table 1:** General characteristics related to gender, age, marital status, and self-reported gingival bleeding between control (GC) and Charcot-Marie-Toothtype2 (GCMT2).

Classification	GC	GCMT2
Gender		
Male	10 (47.6%)	09 (50.0%)
Female	11 (52.4%)	09 (50.0%)
Age	24.42 ± 7.80	27.72 ± 14.51
Marital status		
Single	14 (66.6%)	11 (61.1%)
Married	07 (33.3%)	07 (38.9%)
Gingival bleeding		
No	11 (52.4%)	09 (50.0%)
Yes	10 (47.6%)	09 (50.0%)

**Table 2:** Mean values of decayed, missing, and filled teeth (DMFT) between the groups.

Indicator	GC		GCMT2	
	Mean	SD	Mean	SD
Decayed	2.23	2.99	2.22	2.21
Missing	2.33	2.37	1.78	3.09
Filling	2.80	2.69	1.55	2.95
DMFT	2.46	2.67	1.85	2.74

**Table 3:** Distribution of mean values of salivary parameters between groups.

Variable	GC (Mean ± SD)	GCMT2 (Mean ± SD)
Total salivary volume of salivary flow	9.14 ± 2.74	10.23 ± 3.17
	1.83 ± 0.55	2.05 ± 0.63
pH	7.00 ± 0.57	7.13 ± 0.94
Buffer capacity	98.57 ± 34.53	86.11 ± 34.66

**Table 4:** Distribution of temporomandibular disorder according to self-reported symptoms between GC and GCMT2.

	GC (*n* = 21)		GCMT2 (*n* = 18)	
	*N*	%	*N*	%
Free TMD	14	66.67	11	61.11
Mild TMD	6	28.57	4	22.22
Moderate TMD	1	4.76	3	16.67
Severe TMD	0	—	0	—

**Table 5:** Distribution of clinical signs of TMD and sleep bruxism between groups.

Clinical signs, TMD	GC (*n* = 21)		GC (*n* = 21)		*P* value
	*N*	%	*N*	%	
Mandibular motion					
Straight	15	71.43	15	83.33	0.465^a^
Deviation	6	28.58	3	16.67	
Range of mandibular movements					
Mouth opening (<40 mm)	1 (51.4 ± 6.57)	4.76	2 (47.6 ± 6.30)	11.11	0.586^a^
Lateral excursion to the right (<7 mm)	4 (8.09 ± 2.36)	19.04	5 (8.5 ± 3.40)	27.77	0.706^a^
Lateral excursion to the left (<7 mm)	4 (8.04 ± 2.39)	19.04	3 (9.5 ± 3.56)	16.67	1.000^a^
TMJ sounds					
Mouth opening	1	4.76	4	22.22	0.1618^a^
Mouth closing	1	4.76	4	22.22	0.1618^a^
Temporalis muscle pain					
Anterior	3	14.28	3	16.67	—
Medium	1	4.76	5	27.77	—
Posterior	2	9.52	2	11.11	—
Total	4	19.04	6	33.33	0.465^a^
Mild degree	3	14.28	5	27.77	—
Moderate degree	0	—	1	5.55	—
Severe degree	1	4.76	0	—	—
Masseter muscle pain					
Masseter origin	4	19.04	8	44.44	—
Masseter body	4	19.04	11	61.11	—
Masseter insertion	4	19.04	7	38.88	—
Total	8	38.09	12	66.67	0.075^b^
Mild degree	7	33.33	8	44.44	—
Moderate degree	0	—	4	22.22%	—
Severe degree	1	4.76%	0	—	—
Sleep bruxism	1	4.76%	4	22.22%	0.162^a^

^a^Fisher Exact Test, ^b^chi-square test.
